# The relationship between anticipatory grief and illness uncertainty among Chinese family caregivers of patients with advanced lung cancer: a cross-sectional study

**DOI:** 10.1186/s12904-022-00925-4

**Published:** 2022-03-08

**Authors:** Jiaojiao Li, Di Sun, Xu Zhang, Lihua Zhao, Yanling Zhang, Hongmei Wang, Ni Ni, Guichun Jiang

**Affiliations:** 1 Department of Thoracic Medicine, Cancer Hospital of China Medical University, Liaoning Cancer Hospital & Institute, No.44, Xiaoheyan Road, Shenyang, 110000 Liaoning China; 2 School of Nursing, Liaoning University of Traditional Chinese Medicine, No.79, Chongshan East Road, Shenyang, 110000 Liaoning China; 3 School of Nursing, China Medical University, No.77 Puhe Road, Shenyang, 110000 Liaoning China; 4 School of Nursing, Dalian Medical University, No.9 West Section Lvshun South Road, Dalian, 116044 Liaoning China; 5 Department of Gastroenterology, Cancer Hospital of China Medical University, Liaoning Cancer Hospital & Institute, No.44, Xiaoheyan Road, Shenyang, 110000 Liaoning China; 6 Department of Nursing, Cancer Hospital of China Medical University, Liaoning Cancer Hospital & Institute, No.44, Xiaoheyan Road, Shenyang, 110000 Liaoning China

**Keywords:** Lung cancer, Anticipatory grief, Illness uncertainty, Family caregivers

## Abstract

**Background:**

Anticipatory grief has been shown to be highly prevalent among family caregivers of patients with advanced illness. Qualitative study suggests that illness uncertainty may be one of the core characteristics of anticipatory grief, but it has not been confirmed in quantitative studies. Therefore, the purpose of this study was to explore the relationship between anticipatory grief and illness uncertainty among Chinese family caregivers of patients with advanced lung cancer and to determine the factors influencing anticipatory grief.

**Methods:**

This descriptive cross-sectional study used a convenience sampling method and recruited 254 inpatient family caregivers from the thoracic medicine ward of Liaoning Cancer Hospital & Institute in Shenyang, mainland China. Anticipatory grief (Anticipatory Grief Scale (AGS), illness uncertainty (Uncertainty in Illness Scale Family Caregiver Version) and sociodemographic information (Self-compiled general information questionnaire) were measured using validated self-report measures.

**Results:**

Chinese family caregivers of patients with advanced lung cancer had high levels of anticipatory grief (73.5 ± 16.1). The results of the correlation analysis showed a positive association between anticipatory grief and illness uncertainty (*r* = 0.580, *P* < 0.001). The final linear regression model with anticipatory grief as the dependent variable included four variables: illness uncertainty (β = 0.674, *P* < 0.001), lack of informativeness (β =  − 0.168, *P* = 0.08), monthly income (β = 0.139, *P* = 0.006), and caregiving burden (β =  − 0.196, *P* < 0.001).

**Conclusions:**

Illness uncertainty is probably an important factor affecting anticipatory grief. Excessive caregiving burden is associated with high levels of anticipatory grief. Improving illness uncertainty and caregiving burden may effectively reduce anticipatory grief among Chinese family caregivers.

## Introduction

Cancer is one of the major diseases that seriously endangers human health, and its incidence is increasing. A total of 19.29 million new cancer cases were reported worldwide in 2020. The number of new cancer cases worldwide is expected to exceed 27 million by 2040, while the number of cancer deaths worldwide is almost 10 million, which means that 1/8 of men and 1/11 of women worldwide will die of cancer [1]. Although the number of new cases of lung cancer has fallen to second place according to the Global Cancer Statistics 2020, there are still 2.21 million new cases of lung cancer accounting for 11.4% of all new cancer cases worldwide. Lung cancer remains the leading cause of cancer death, with an estimated 1.8 million deaths (18%). In China, there were approximately 787,000 new cases of lung cancer and 631,000 deaths in 2015, and the diagnosis often occurs so late that approximately two-thirds of patients have lost the opportunity for radical surgery [2, 3]. Patients with advanced cancer are often more prone to side effects such as nausea, vomiting, fatigue and malnutrition, which affects the patient’s quality of life and places a heavy burden on family caregivers in terms of time, energy and finances [4, 5]. The family caregiver (FC) definition includes any family member, friend, or partner who maintains a significant relationship with the patient and provides some care [6]. FCs, as a vital part of the patient’s social support system, need to undertake a variety of stresses such as the patient’s deteriorating condition, medical decisions, and worries about the future, which can easily trigger a series of physiological and psychological stress reactions. Therefore, along with the progressive deterioration of the patient’s physical and mental condition, FCs must manage expectations and emotions associated with the fear of losing their significant other, a phenomenon designated anticipatory grief (AG) [6, 7].

Lindeman first introduced the concept of AG in 1944, suggesting that AG is the individual’s grief response prior to the period of mourning and that it is an inevitable and important part of the end-of-life grief experience [8]. Subsequently, Rando defined AG as the process of mourning, coping, planning, and psychosocial restructuring and their interactions triggered by the stimulus of loss. AG is a clinical symptom, and a process with different stages [9]. Currently, a definition for anticipatory grief is not clear. Recent results suggest that AG in family caregivers is defined as the family response to the perceived threat to the other’s life and the subsequent anticipation of loss in the context of the end-of-life caregiving relationship [6]. AG is one of the common psychological stress reactions of FCs of cancer patients prior to the loss of a relative and is at a high level. A study by Nielsen MK and colleagues of 3635 caregivers of terminally ill patients (89% suffered from cancer) showed that approximately one-third of FCs had AG, with up to 15% of those with severe symptoms [10]. AG can cause physical and mental discomfort in the FCs of cancer patients, including depression, despair, pain, sleep disturbance, and loss of appetite. It can also adversely affect the behaviour and social function of family members, and even lead to excessive behaviours such as self-harm and suicide. Current studies have established that anticipatory grief may be associated with caregiver age, education, anxiety, depression, caregiving burden, coping style, and social support [11–13]. In a recent qualitative study, illness uncertainty was identified as one of the core characteristics in the conceptual framework of AG for family caregivers. Illness uncertainty may cause several FCs to develop into a permanent state of hypervigilance towards illness signs, mainly after crisis episodes. Caregiving in life-threatening conditions causes FCs to produce traumatic distress, which, as one of the main dimensions of AG, can manage the threat to the other’s life [6]. Illness uncertainty is defined as “the inability to determine the meaning of illness-related events and accurately anticipate or predict health outcomes” [14]. Previous findings suggest that FCs of cancer patients have high levels of illness uncertainty. Illness uncertainty among family caregivers due to a lack of valid information may increase FCs’ mental stress, leading to reduced caregiving capacity and affecting patients’ recovery from illness [15–17]. However, few quantitative studies have focused on the relationship between illness uncertainty and anticipatory grief among FCs. Given the high prevalence and potential adverse outcomes of these two factors among FCs of cancer patients, a more in-depth examination of the relationship is warranted to inform the development of precise interventions to improve negative emotions in FCs.

The purpose of this study was to determine the occurrence of anticipatory grief and illness uncertainty among Chinese family caregivers of patients with advanced lung cancer. In addition, multivariate techniques were used to identify factors associated with anticipatory grief and the relationship between anticipatory grief and illness uncertainty.

## Methods

### Study design and participants

This descriptive cross-sectional study used a convenience sampling method and recruited inpatient family caregivers from the thoracic medicine ward of Liaoning Cancer Hospital & Institute in Shenyang, mainland China, between January and May 2021. The criteria for inclusion of participants in this study were as follows: 1) family caregivers of patients with a clinicopathological histological or cytological diagnosis of lung cancer and TNM stage III or IV; 2) knowledge of the patient’s disease condition; 3) age ≥ 18 years; 4) good reading and communication skills in Chinese; and 5) assumption of primary care of the patient’s daily life and identified by the patient as his or her primary caregiver. The exclusion criteria were as follows: 1) the patient’s family had mental, psychological, or cognitive impairment; and 2) the length of care was less than 1 week.

### Ethics

This study was granted ethical approval by the Liaoning Cancer Hospital & Institute Ethics Committee. Participants were informed of the purpose and procedures of the study prior to the start of the study per the Declaration of Helsinki. They had the right to leave the study at any time and were not required to answer any questions. Informed written consent was obtained from all participants prior to participation.

### Procedure

The questionnaire was distributed by members of the study team and completed independently by family caregivers of patients with advanced lung cancer. Prior to completing the questionnaire, study team members used a uniform instruction guide to introduce the purpose and meaning of the survey to study participants and assist participants who had difficulty reading the questionnaire. The questionnaire consisted of three main self-reported sections: general demographic characteristics of the patient and family caregiver, the Uncertainty in Illness Scale Family Caregiver, and the Anticipatory Grief Scale. The participants completed the questionnaires and collected by the research team members on the spot, and the participants were asked to complete any missing options. All participants were given a small gift after completing the questionnaire as compensation for their time spent completing the questionnaire. The study was reported following the STROBE guidelines.

### Measures

#### Self-compiled general information questionnaire

This questionnaire includes FCs demographic data (e.g., age, gender, monthly income, education level, etc.) and patients’ clinical data which were obtained from medical records (e.g., patient age, metastasis or recurrence, number of hospitalizations for cancer treatment, etc.).

#### Uncertainty in illness scale family caregiver version

The scale was developed in the United States by Professor Mishel to measure the FC’s level of uncertainty in illness [18]. The Chinese version had a cross-cultural adaptation, facial validity, content validity, and internal consistency [19]. The scale is divided into 4 dimensions: ① unpredictability, which refers to the lack of contingency between illness and treatment cues and illness outcome (4 items); ② Lack of informativeness refers to the lack of information related to the diagnosis and severity of the disease (5 items); ③ complexity: the cues about the treatment and the system of care are multiple, intricate and varied (8 items); ④ ambiguity, which refers to address clues about the state of the illness, such as being able to determine whether the illness is getting better or worse (13 items). All 30 items are summed to calculate a total score that ranges from 30 to 150, with higher scores indicating higher levels of illness uncertainty. The content validity index (CVI) was 0.87, and Cronbach’s α was 0.89 [19]. Cronbach’s alpha in the current sample was 0.79.

#### Anticipatory grief scale (AGS)

The scale was developed by Theut et al. and was used to measure anticipatory grief [20]. The AGS is a 27-item measure of anticipatory grief scored on a 5-point Likert scale. Scores range from 5 to 135, with higher scores being indicative of higher levels of anticipatory grief [21]. The scale has been applied to populations of patient guardians, and caregivers of patients with dementia or cancer, and it typically takes approximately 10–15 min to complete [11, 22]. This study used the Chinese version of the AGS scale, which has seven dimension: anger, guilt, anxiety, irritability, grief, feelings of loss, and decreased ability to function at usual tasks. The Chinese version of the AGS scale was cross-culturally adapted, with a content validity of 0.96 and Cronbach’s alpha 0.90 [23]. The Chinese version of the AGS has good reliability and validity. The entries are easy to understand and answer, and health care workers and volunteers can understand the feelings of caregivers through the scale. Cronbach’s alpha in the current sample was 0.92.

### Statistical analysis

The sociodemographic characteristics of the participants were examined by computing frequencies, percentages, means, and standard deviations. The Kolmogorov–Smirnov test was used to examine whether the assumption of normality was met and, consequently, ascertain the appropriateness of parametric analyses. One-way ANOVAs and t tests were used to determine factors associated with anticipatory grief among FCs initially, and Pearson’s r correlations were used to test for unadjusted associations between variables. For regression models, AGS scores were normally distributed, and residual plots and tests for equality of variance demonstrated no major departure from homoscedasticity, indicating that linear regression is appropriate [24]. Multivariate linear stepwise regression was used to analyse the influencing factors of anticipatory grief. The unstandardized coefficient (B), standard error (SE), standardized coefficient (b), t statistic, *p* value and 95% confidence intervals are reported. All statistical tests were two-tailed and statistical significance for all analyses was set at 0.05. The results were analysed using SPSS Statistics version 26.0 (IBM Corp., Armonk, NY, 237 USA).

## Results

### Descriptive statistics

A total of 254 FCs of patients with advanced lung cancer participated in this study. There were 110 males (43.3%) and 144 females (56.7%) in the sample. The age of the FCs was mainly 36–59 years old (*n* = 150; 59.1%), and most of the FCs were spouses (*n* = 89; 35.0%) or parents (*n* = 136; 53.5%) of the patients.

The results of the statistical analysis of baseline characteristics and anticipatory grief scores of family caregivers and patients are shown in Table 1. FCs aged ≤ 35 years reported significantly higher AGS scores than those aged 36–60 years and ≥ 60 years (*F* = 5.649, *P* < 0.05). FCs with a bachelor’s degree and above reported higher AGS scores than those with high school education, primary school education and below (*F* = 3.404, *P* < 0.05). Regarding monthly income, FCs with less income reported higher AGS scores than those with more income (*F* = 3.255, *P* < 0.05). FCs who were divorced or widowed reported higher AGS scores than those who were married and unmarried (*F* = 6.217, *P* < 0.05). Regarding relationships with patients, children reported lower AGS scores than compared with spouses, parents and others (*F* = 12.226, *P* < 0.001). FCs without the experience of bereavement reported higher AGS scores than those with the experience of bereavement (*t* = 2.945, *P* < 0.05). There were significant differences in the AGS scores for FCs with different lengths of care (*F* = 3.319, *P* < 0.05). FCs with severe caregiving burden reported higher AGS scores than those with moderate, mild and no caregiving burden (*F* = 7.456, *P* < 0.001). FCs with a family history of malignancy reported higher AGS scores than those without a family history of malignancy (*t* = 2.616, *P* < 0.05).

**Table 1:** Baseline characteristics of family caregivers and patients with statistical analysis of anticipatory grief scores(*n* = 254)

Variable					Statistic	
**Family caregiver data**		*n*	%	Anticipatory grief score (mean ± SD)	*F*/*t*	*P*
Gender	Male	110	43.3%	75 ± 14.2	1.363	0.174
	Female	144	56.7%	72.3 ± 17.3		
Age	≤ 35	71	28.0%	77.7 ± 16.9	**5.649**	**0.004**
	36–59	150	59.1%	73 ± 15.3		
	≥ 60	33	13.0%	66.7 ± 15.7		
Education level	Primary school and below	10	3.9%	71.1 ± 15.5	**3.404**	**0.035**
	High school	134	52.8%	71.2 ± 16		
	Bachelor and above	110	43.3%	76.5 ± 15.9		
Monthly income	> 6000	64	25.2%	68.5 ± 16.6	**3.255**	**0.022**
	4000–6000	79	31.1%	73.6 ± 15.7		
	2000–4000	61	24.0%	75.9 ± 14.9		
	< 2000	50	19.7%	76.8 ± 16.4		
Marital status	Unmarried	22	8.7%	81.7 ± 17.9	**6.217**	**0.002**
	Married	218	85.8%	72.1 ± 15.8		
	Divorced or widowed	14	5.5%	82.6 ± 10.9		
Relationship with patients	Spouse	89	35.0%	66.9 ± 15.3	**12.226**	< **0.001**
	Parents	136	53.5%	77.1 ± 14.8		
	Children	11	4.3%	65.3 ± 14.4		
	Others	18	7.1%	84.2 ± 17.2		
Religious	No	236	92.9%	73.7 ± 16.2	0.806	0.421
	Yes	18	7.1%	70.6 ± 14.4		
The experience of bereavement	No	176	69.3%	75.5 ± 15.9	**2.945**	**0.004**
	Yes	78	30.7%	69.1 ± 15.8		
Length of care	< 3 months	95	37.4%	75.5 ± 15.8	**3.319**	**0.021**
	3–6 Months	54	21.3%	75.1 ± 13.4		
	6–12 Months	35	13.8%	66 ± 18.3		
	> 12 Months	70	27.6%	73.2 ± 16.5		
Caregiving burden	Severe	92	36.2%	77.7 ± 17.6	**7.456**	< **0.001**
	Moderate	71	28.0%	74.5 ± 13.9		
	Mild	50	19.7%	72.2 ± 11.8		
	No	41	16.1%	64.1 ± 17		
Family history of malignancy	Yes	52	20.5%	77.7 ± 11.6	**2.616**	**0.010**
	No	202	79.5%	72.4 ± 16.9		
**Patient data**						
Age	≤ 35	4	1.6%	77.3 ± 20.3	0.511	0.601
	36–59	117	46.1%	72.5 ± 16.6		
	≥ 60	133	52.4%	74.3 ± 15.6		
Self-care ability	Fully	174	68.5%	74.3 ± 17.1	0.883	0.415
	Partially	74	29.1%	72 ± 13.8		
	Not	6	2.4%	68.3 ± 12		
Recurrence or metastasis	Yes	218	85.8%	73.7 ± 16.3	0.392	0.695
	No	36	14.2%	72.5 ± 15		
Number of hospitalizations	1 time	61	24.0%	72.3 ± 13.7	1.947	0.145
	2–3 Times	73	28.7%	76.6 ± 14.8		
	> 3 Times	120	47.2%	72.2 ± 17.8		

Independent sample t-test and ANOVA analysis were used to compare the mean score of anticipatory grief

### Correlation between anticipatory grief and illness uncertainty among FCs

Pearson’s correlation analysis (Table 2) showed positive correlations between FCs’ anticipatory grief and total illness uncertainty scores (*r* = 0.580, *P* < 0.001) and the dimensions of ambiguity (*r* = 0.578, *P* < 0.001), complexity (*r* = 0.474, *P* < 0.001), and lack of informativeness (*r* = 0.282, *P* < 0.001). However, no relationship was found between anticipatory grief and the unpredictability dimension (*P* = 0. 736 > 0.05).

**Table 2:** Correlation between anticipatory grief and illness uncertainty among family caregivers

	Anticipatory grief	Grief	Feelings of loss	Anger	Irritability	Guilt	Anxiety	Decreased ability to function at usual tasks
illness uncertainty	0.580**	0.471**	0.180**	0.498**	0.545**	0.508**	0.531**	0.449**
unpredictability	-0.021	-0.073	-0.108	0.106	0.049	0.033	-0.007	-0.088
ambiguity	0.578**	0.510**	0.268**	0.416**	0.457**	0.490**	0.529**	0.468**
complexity	0.474**	0.399**	0.194**	0.448**	0.439**	0.390**	0.394**	0.334**
lack of informativeness	0.282**	0.151**	-0.116	0.299**	0.440**	0.270**	0.290**	0.261**

^**^P < 0.001

### Multiple linear regression analysis for anticipatory grief

Taking anticipatory grief as the dependent variable, the statistically significant variables in Tables 1 and 2 were included as independent variables in the regression equation for analysis. The model was statistically significant (adjusted *R*^2^ = 0.414, *F* = 45.632, *P* < 0.001). Illness uncertainty (β = 0.674, *P* < 0.001), caregiving burden (β =  − 0.196, *P* < 0.001), monthly income (β = 0.139, *P* = 0.006) and lack of informativeness (β =  − 0.168, *P* = 0.08) were the influencing factors of anticipatory grief (Table 3).

**Table 3:** Multiple regression analyses predicting Anticipatory grief (*N* = 254)

Dependent variable	Independent variable	B	SE	β	*t*	*P*
Anticipatory grief	Constant	2.934	7.053		.416	.678
	illness uncertainty	1.026	.097	.674	10.603	.000
	Caregiving burden	-2.906	.736	-.196	-3.948	.000
	Monthly income	2.096	.750	.139	2.795	.006
	lack of informativeness	-1.034	.390	-.168	-2.655	.008

*R* = 0.650, *R*^2^ = 0.423, Adjusted *R*^2^ = 0.414, *F* = 45.632, *P* < 0.001

## Discussion

To the best of our knowledge, the present study may be the first to investigate the relationship between anticipatory grief and illness uncertainty among FCs of patients with advanced lung cancer. The findings showed that FCs had an anticipatory grief score of 73.5 ± 16.1. Although the Anticipatory Grief Scale does not identify a diagnostic threshold, the scores derived from this study were close to the median score of 81 on the Anticipatory Grief Scale, which may suggest a moderate level of anticipatory grief among family caregivers in general. In addition, the results found that illness uncertainty, caregiving burden, monthly income, and lack of information may be influential factors in anticipatory grief among FCs. The predictor of anticipatory grief according to the β-value may be illness uncertainty.

Illness uncertainty is a cognitive state that inhibits the person from determining the meaning of the events related to the disease, which generates an inability to assign definite values to objectives and events and to predict consequences accurately [14]. The present study found high levels of (86.02 ± 10.57) illness uncertainty among FCs of patients with advanced lung cancer. Other studies have confirmed that family caregivers of patients receiving palliative care have higher levels of uncertainty than family caregivers of patients with other types of acute or chronic illness [16, 25]. Illness uncertainty can further increase the physical and psychological stress of family caregivers, leading to fatigue, depression, anxiety, isolation, loneliness, grief, and depression, resulting in lower quality of care, which can affect the patient’s recovery [26, 27]. Furthermore, based on the linear regression model constructed in this study, illness uncertainty and lack of information dimensions were identified as influencing factors of anticipatory grief. This may be because FCs of palliative care patients not only need to care for the patients’ daily life but also manage complex equipment such as drainage systems or infusion pumps and the application of pain medications are becoming an important part of care as the patients’ disease progresses [28]. FCs are faced with increasing patient care needs that may trigger the illnesses uncertainty of patients and FCs if they are not supported by ongoing information from health professionals. At the same time, the threat of death that often accompanies patients receiving palliative care leads family caregivers to face the risk of losing a family member.This makes the experience of caring for a patient in palliative care unique and complex, ultimately leading to anticipatory grief for FCs [29, 30]. Therefore, based on the findings of this study, it appears that anticipatory grief can be reduced by improving the uncertainty of illness in FCs. However, there is a paucity of research on the relationship between anticipatory grief and illness uncertainty in FCs. More high-quality research is needed clarify the relationship further and construct evidence-based interventions to avoid anticipatory grief.

Two additional influences on anticipatory grief in family caregivers were found to be caregiving burden and family caregiver monthly income. In other studies of family caregivers of patients with advanced Parkinson’s and Alzheimer’s disease, caregiving burden was also confirmed to be positively associated with anticipatory grief. Financial status was also an important factor in the negative emotions and perceptions of family caregivers [31–33]. These results suggest that anticipatory grief may be present in almost all family caregivers of patients with advanced disease.

Influenced by traditional Chinese Confucian culture and filial piety, caring for an ill loved one is considered every family member’s responsibility. In addition, but in China, the family is also an integral part of treatment decisions [34]. Together, these reasons lead to a heavy caregiving burden for Chinese FCs responsible for almost all of the caregiving tasks and stresses of terminally ill cancer patients. Caregiving burden overload has been shown to be significantly associated with anxiety, depression, and many other negative emotions, and anticipatory grief may be one of them in conjunction with the results of this study [35]. In China, it is believed that the topic of death may affect the patient’s emotions and bring misfortune. As a result, the grief felt by family caregivers prior to the death of a cancer patient, despite the burden of care, is usually not publicly acknowledged, publicly mourned, or socially supported [32, 36]. This is the most important barrier to improving the quality of a good death for Chinese patients and may be the root cause of anticipatory grief. Based on the results of this study, family caregivers with low income may have higher levels of anticipatory grief. Similar results have been obtained from a previous study [37]. However, we believe that income may not directly influence anticipatory grief, but rather is mediated through caregiver burden. It seems easy to understand that family caregivers with low income often cannot afford to hire others to help care for the patien. The heavy caregiving tasks usually requirse the family caregiver to carry them alone, which undoubtedly creates a heavy caregiving burden. In advanced cancer, poor patient physical and mental health is associated with higher caregiver burden regardless of hours caregiving [38]. Therefore, improving family caregiver burden may have a positive effect on reducing anticipatory grief, suggesting that practical and effective interventions targeting family caregiver caregiving burden remain a focus of future research attention.

The present study also has some limitations. First, this study is a cross-sectional study. It is not yet possible to determine the causal relationship between the influential factor variables found in the study and anticipatory grief. Future large sample cohort studies are needed to validate these findings further. Second, the primary purpose of this study was to determine the relationship between illness uncertainty and anticipatory grief. Thus, the caregiving burden of FCs was investigated using a self-report format rather than an objective caregiving burden scale. This may not fully reflect the caregiving burden, as it only represents the caregiver’s perception. Finally, we used a convenience sample of FCs that, by being a nonprobability sampling method, may not adequately represent the population.

## Conclusions

Anticipatory grief is prevalent among Chinese family caregivers of patients with advanced cancer, and anticipatory grief may be influenced by factors such as uncertainty about the disease, lack of information, monthly income, and caregiving burden. Illness uncertainty is probably an important factor affecting anticipatory grief. Family caregivers with low monthly income and high caregiving burden exhibited higher levels of anticipatory grief. Further studies should continue to explore modifiable risk and protective factors related to the family caregivers’ anticipatory grief to improve services for this population.

## Data Availability

All data sets on which the conclusions of the paper are based are available upon request to the corresponding author. All methods were performed in accordance with the relevant guidelines and regulations.

## References

[1] Sung H, Ferlay J, Siegel RL, Laversanne M, Soerjomataram I, Jemal A (2021). Global Cancer Statistics 2020: GLOBOCAN Estimates of Incidence and Mortality Worldwide for 36 Cancers in 185 Countries. CA Cancer J Clin.

[2] Chen W, Zheng R, Baade PD, Zhang S, Zeng H, Bray F (2016). Cancer statistics in China, 2015. CA Cancer J Clin.

[3] Hong Q, Wu G, Qian G, Hu C, Zhou J, Chen L (2015). Prevention and management of lung cancer in China. Cancer-Am Cancer Soc.

[4] Henson LA, Maddocks M, Evans C, Davidson M, Hicks S, Higginson IJ (2020). Palliative Care and the Management of Common Distressing Symptoms in Advanced Cancer: Pain, Breathlessness, Nausea and Vomiting, and Fatigue. J Clin Oncol.

[5] Alam S, Hannon B, Zimmermann C (2020). Palliative Care for Family Caregivers. J Clin Oncol.

[6] Coelho A, de Brito M, Teixeira P, Frade P, Barros L, Barbosa A (2020). Family Caregivers’ Anticipatory Grief: A Conceptual Framework for Understanding Its Multiple Challenges. Qual Health Res.

[7] Wittenberg-Lyles E, Demiris G, Oliver DP, Washington K, Burt S, Shaunfield S (2012). Stress variances among informal hospice caregivers. Qual Health Res.

[8] Lindemann E (1994). Symptomatology and management of acute grief, 1944. Am J Psychiatry.

[9] Rando TA (1988). Anticipatory grief: the term is a misnomer but the phenomenon exists. J Palliat Care.

[10] Nielsen MK, Neergaard MA, Jensen AB, Bro F, Guldin M (2016). Psychological distress, health, and socio-economic factors in caregivers of terminally ill patients: a nationwide population-based cohort study. Support Care Cancer.

[11] Burke LA, Clark KA, Ali KS, Gibson BW, Smigelsky MA, Neimeyer RA (2015). Risk Factors for Anticipatory Grief in Family Members of Terminally Ill Veterans Receiving Palliative Care Services. J Soc Work End Life Palliat Care.

[12] Tomarken A, Holland J, Schachter S, Vanderwerker L, Zuckerman E, Nelson C (2008). Factors of complicated grief pre-death in caregivers of cancer patients. Psychooncology.

[13] Hudson PL, Thomas K, Trauer T, Remedios C, Clarke D (2011). Psychological and social profile of family caregivers on commencement of palliative care. J Pain Symptom Manag.

[14] Mishel MH (1988). Uncertainty in illness. Image J Nurs Sch.

[15] Taylor J, Fradgley E, Clinton-McHarg T, Byrnes E, Paul C (2021). What are the sources of distress in a range of cancer caregivers? A qualitative study. Support Care Cancer.

[16] Arias-Rojas M, Carreño-Moreno S, Posada-López C (2019). Uncertainty in illness in family caregivers of palliative care patients and associated factors. Rev Lat-Am Enferm..

[17] Shilling V, Starkings R, Jenkins V, Fallowfield L (2017). The pervasive nature of uncertainty-a qualitative study of patients with advanced cancer and their informal caregivers. J Cancer Surviv.

[18] Mishel M (1997). Uncertainty in illness scales manual.

[19] Hongyan C: The relationship among uncertainty in illness, social support and coping style about family members of patients with chronic disease. Master Thesis, Yanbian University; 2010. (in Chinese)

[20] Theut SK, Jordan L, Ross L (1991). Caregiver's Anticipatory Grief in Dementia: A Pilot Study. Int. J. Aging Hum. Dev.

[21] Glick DR, Motta M, Wiegand DL, Range P, Reed RM, Verceles AC (2018). Anticipatory grief and impaired problem solving among surrogate decision makers of critically ill patients: A cross-sectional study. Intens Crit Care Nur.

[22] Fowler NR, Hansen AS, Barnato AE, Garand L (2013). Association between anticipatory grief and problem solving among family caregivers of persons with cognitive impairment. J Aging Health.

[23] Dajun X: Study on grief of advanced cancer patients and their families. Master Thesis, Southwest Medical University; 2016. (in Chinese)

[24] Bresenham D, Kipp AM, Medina-Marino A (2020). Quantification and correlates of tuberculosis stigma along the tuberculosis testing and treatment cascades in South Africa: a cross-sectional study. Infect Dis Poverty.

[25] Northouse LL, Mood DW, Montie JE, Sandler HM, Forman JD, Hussain M (2007). Living with prostate cancer: patients' and spouses' psychosocial status and quality of life. J Clin Oncol.

[26] Arias-Rojas M, Carreño-Moreno S, Rojas-Reyes J (2020). Uncertainty Towards the Disease of Family Caregivers of Patients in Palliative Care: A Scoping Review. Aquichan.

[27] Haley WE, LaMonde LA, Han B, Narramore S, Schonwetter R (2001). Family caregiving in hospice: effects on psychological and health functioning among spousal caregivers of hospice patients with lung cancer or dementia. Hosp J.

[28] Hudson PL, Remedios C, Thomas K (2010). A systematic review of psychosocial interventions for family carers of palliative care patients. Bmc Palliat Care.

[29] Candy B, Jones L, Drake R, Leurent B, King M (2011). Interventions for supporting informal caregivers of patients in the terminal phase of a disease. Cochrane Database Syst Rev.

[30] Zavagli V, Raccichini M, Ercolani G, Franchini L, Varani S, Pannuti R (2019). Care for Carers: an Investigation on Family Caregivers' Needs, Tasks, and Experiences. Transl Med UniSa.

[31] Fox S, Azman A, Timmons S (2020). Palliative care needs in Parkinson’s disease: focus on anticipatory grief in family carers. Ann Palliat Med.

[32] Cheung DSK, Ho KHM, Cheung TF, Lam SC, Tse MMY (2018). Anticipatory grief of spousal and adult children caregivers of people with dementia. Bmc Palliat Care.

[33] Lindeza P, Rodrigues M, Costa J, Guerreiro M, Rosa MM (2020). Impact of dementia on informal care: a systematic review of family caregivers’ perceptions. Bmj Support Palliat.

[34] Tang Y (2019). Caregiver burden and bereavement among family caregivers who lost terminally ill cancer patients. Palliat Support Care.

[35] Perpiñá-Galvañ J, Orts-Beneito N, Fernández-Alcántara M, García-Sanjuán S, García-Caro MP, Cabañero-Martínez MJ (2019). Level of Burden and Health-Related Quality of Life in Caregivers of Palliative Care Patients. Int J Env Res Pub He.

[36] Gu X, Cheng W, Cheng M, Liu M, Zhang Z (2015). The preference of place of death and its predictors among terminally ill patients with cancer and their caregivers in China. Am J Hosp Palliat Care.

[37] Li J, Li Y, Li P (2021). Perceived Grief Among Caregivers of Patients With Dementia in China. Clin Nurs Res.

[38] Semere W, Althouse AD, Rosland AM, White D, Schenker Y (2021). Poor patient health is associated with higher caregiver burden for older adults with advanced cancer. J Geriatr Oncol.

